# Heavy Metal and Metalloid Contamination Assessments of Soil around an Abandoned Uranium Tailings Pond and the Contaminations’ Spatial Distribution and Variability

**DOI:** 10.3390/ijerph15112401

**Published:** 2018-10-29

**Authors:** Wei-hong Wang, Xue-gang Luo, Zhe Wang, Yu Zeng, Feng-qiang Wu, Zhong-xiang Li

**Affiliations:** 1 College of Environment and Resources, Southwest University of Science and Technology, Mianyang 621010, China; wangzhe@swust.edu.cn (Z.W.); zengyu92@gmail.com (Y.Z.); wufengqiang@swust.edu.cn (F.-q.W.); lizhongxiang@swust.edu.cn (Z.-x.L.); 2 State Key Laboratory of NBC Protection for Civilian, Beijing 102205, China; 3 Engineering Research Center of Biomass Materials, Ministry of Education, Mianyang 621010, China

**Keywords:** heavy metal and metalloid pollution assessment, comprehensive pollution index (CPI), uranium tailings pond, spatial distribution, spatial variability

## Abstract

To investigate the heavy metal and metalloid contamination of soil around a Huanan uranium tailings pond, abandoned in 1998, we defined a study area of 41.25 km^2^ by a natural boundary and targeted 5 elements’ (U, Mn, As, Pb, Cr) single contamination and comprehensive pollution as the assessment contents. First, we collected 205 samples and evaluated them with the contamination factor (*CF*) method aiming at judging whether the single target element concentration exceeded the local background value and environmental quality standard. We obtained *CF*_1_ (the background value of a certain target element as the baseline value) and *CF*_2_ (the environmental quality standard for soils as the baseline value). Second, we evaluated the ecological risk of the key pollutant U with the risk assessment code (RAC) method, taking the 27 samples whose *CF*_2_ > 1 as examples and concluded that the environmental risk of U was relatively high and should arouse concern. Third, we selected comprehensive pollution index (CPI) to assess the compound pollution degree of five target elements. Fourth, we constructed the U contamination and CPI’s continuous distribution maps with spatial interpolation, from which we worked out the sizes and positions of slightly, moderately and strongly polluted zones. Finally, we analyzed the spatial variability of U and CPI with the aid of a geostatistical variogram. We deduced that the spatial variation of uranium was in close relationship with local topography, and probably precipitation was the driving force of U contamination diffusion, whereas CPI exhibited weak spatial dependence with random characteristics. The above work showed that 3.14 km^2^ soil near the pond was fairly seriously polluted, and the other 4 elements’ single contaminations were less serious, but the 5 target elements’ cumulative pollution could not be ignored; there were other potential pollution sources besides the uranium tailings pond. Some emergency measures should be taken to treat U pollution, and bioremediation is recommended, taking account into U’s high bioavailability. Further, special alerts should be implemented to identify the other pollution sources.

## 1. Introduction

Uranium tailings ponds may contain a large amount of radionuclide and non-radioactive potentially hazardous elements and cause contamination of the surrounding soil and water environment [1]. The pollution of uranium in the surrounding soil of uranium mining and metallurgy regions has been reported many times and has attracted much attention [1,2,3,4,5,6,7,8]. Irrational contamination assessments often result in underestimating the pollution hazards or “over protecting” the research areas. For farmland, contamination assessment is furthermore related to the pattern of land use, the selection of crop planting types, and the safety of agricultural products [9].

The Huanan uranium tailings pond, situated in south-central China, is the largest source of radioactive pollution in the uranium mining and metallurgical system of China. Since the served uranium smelting plant was not active, the tailings pond was covered by clay with an average thickness 80 cm and then decommissioned in 1998. Originally, the land around the uranium tailings pond was farm field, but it was not cultivated since the uranium smelting plant was put into operation. With the increasing shortage of land resources, the rational management and use of the soil around the tailings pond are becoming more and more necessary, for which scientific evaluation is a prerequisite.

In order to confirm the potentially hazardous elements of the study area, we referred to Environmental Quality Evaluation Standards for farmland of Edible Agricultural Products of China (HJ/T 332-2006), which regulates that the heavy metal and metalloid elements Cd, Hg, As, Pb, Cr, Cu, Zn, and Ni should be considered to determine whether a piece of land is suitable to be used as farmland for edible agricultural products. We collected 24 samples in advance inside the uranium tailings pond, which showed the concentrations of Cd, Hg, Cu, Zn, and Ni were in line with Chinese national standards, so we excluded these five elements. During operation of the uranium smelting plant, potassium permanganate was heavily used as the catalyzer, so we decided to choose Mn as one of the assessed elements. Finally, 5 heavy metal and metalloid elements (U, Mn, As, Pb, Cr) were targeted in this study. Many experimental reports showed that in low-dose radionuclide-contaminated soil, the chemical toxicity of radionuclides was much stronger than its radiotoxicity, so radioactive toxicity can be negligible [10]. We did not take into account the radioactive pollution in this paper, since the uranium contents of the Huanan tailings pond were 3.21–120.52 μg/g.

With regard to the assessment methods of potentially hazardous element contaminations in soil, there are two kinds—index methods and model index methods. Index methods refer to substituting the actual pollutant concentrations into the mathematical formula to obtain the pollution indices, and then comparing them with the corresponding assessment criteria to determine the level of pollution. The model index methods, based on index methods, assess potentially hazardous element pollutions by constructing complicated mathematical models and have some advantages over index methods when processing the fuzzy boundary effect, but they require a lot of functions and cumbersome operations, and how to determine the optimal weight is a key problem which limits their applications. The index methods are the preferred methods when people evaluate the soil quality. Many scholars have conducted these methods in relevant research [1,11,12].

Through careful identifications and comparisons, this study assessed the sampling points’ single element’s contamination with the contamination factor method aiming at judging whether the single kind of element exceeded the local background value and environmental quality standard, and with the risk assessment code (RAC) method aiming at evaluating the ecological risk of the key pollutant. Then, we selected the comprehensive pollution index (CPI) to assess the compound and cumulative pollution degree of five target elements.

The concentration of elements in soil is a continuous spatial variable and has the characteristics of regionalized variation. The pollution situation varies with the change in spatial position, and the pollution condition of the soil through statistical analyses only directed against the sampling points cannot reflect the spatial distribution characteristics of the whole study area.

To fully determine the pollution degrees of potentially hazardous elements, we explored the spatial characteristics of the regional pollution using spatial interpolation and constructed pollution distribution maps after assessing the discrete sampling points. Furthermore, we explained the spatial variation of uranium and deduced the driving force of uranium pollution diffusion.

In short, the aim of this paper is to assess the contamination degree and scope of the vicinity of the Huanan uranium tailings pond and to evaluate the possibility of restoring cultivation.

## 2. Materials and Methods

### 2.1. Soil Sample Collection and Testing

**Figure 1:**
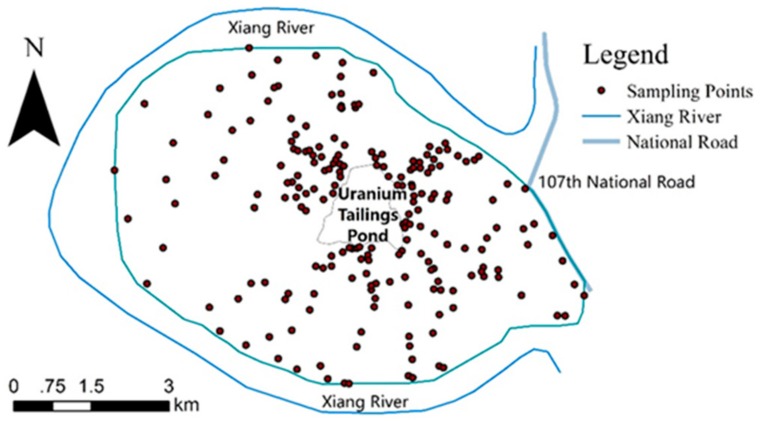
The study area.

We defined the study area, which is 41.25 km^2^, by a natural boundary. The sampling layout was as follows: take the tailings pond as the center, southward, westward, and northward to Xiang River, and eastward 3 km to the 107th National Road. The nearer to the tailings pond, the denser the sampling points. In addition, in places with larger terrain changes, sampling points were added (sample point distribution, see Figure 1). In total, 205 soil samples were collected, with the sampling depth of 30 cm, and GPS RTK communication with the base station was used to record the sampling points’ coordinates and elevations precisely.

After tri-acid (HF-HNO_3_-HCL) digestion, the total U concentrations of all samples were tested with the inductively coupled plasma mass spectrometry (Agilent 7700x, Agilent Technologies, Inc., 9-1 Takakura-cho, Hachioji-shi Tokyo, Japan), and those of the other four elements with the atomic absorption spectrometer (Perkin-Elmer AANALYST 700, Singapore), in the Analysis and Testing Center of Southwest University of Science and Technology. We extracted the acid-soluble fractions (F1) of the 27 samples whose total U concentrations exceeded the standard value with the BCR sequential extraction method (BCR-SEP) and tested the F1’s U contents with the Agilent 7700x ICP-MS in order to evaluate the ecological risk of the key pollutant U with the risk assessment code (RAC) method.

### 2.2. Heavy Metal and Metalloid Pollution Assessment Methods

#### 2.2.1. Summary of Index Methods

As mentioned earlier, the index methods are the preferred methods when people evaluate soil quality, among which the internationally accepted main indices include the contamination factor (CF) [13], geoaccumulation index [13], enrichment factor (EF) [14], risk assessment code (RAC) [15], Nemerow index [16], Pollution Load Index (PLI) [17], the potential ecological risk index [18], mean Effects Range Median quotient (mERMq) [19], and cumulative Normalized and Weighted Average Concentration (c_NWAC) [20] etc. The last two methods were created to assess sediments’ quality making use of cumulative indexes referring to the corresponding datasets, and we excluded them firstly.

The contamination factor is also called the single-factor pollution index; dividing each concentration by a baseline concentration for each chemical, is the basis of other environmental quality indexes, environmental quality classification and comprehensive evaluation. Taking the background value as the baseline value, the contamination factor can be used to reflect the degree of human-caused disturbance on the soil, and if taking the environmental quality standard for soils or soil screening value as the baseline value, it can be used to assess the degree of contamination and evaluate the impact of soil environmental quality on human life [17,21,22].

The geoaccumulation index was originally used to study the pollution degree of river deposits, and it has been also applied to evaluate subsequent soil pollution. Compared with the measured contents of elements in ambient medium and the geochemical background values of target elements, the background value changes from geochemical factors and lithogenesis can be reduced. Nevertheless, there are great differences between soil and sediments in heavy metal and metalloid migration; consequently, soil assessment results according to the river deposit pollution classifying scope are divergent from reality.

The enrichment factor (EF) was created to identify the sources of atmospheric particulates over Antarctica. Thereafter, EF was extended to other fields including soil heavy metal and metalloid assessments. This method standardizes the concentration of samples by selecting a standardized element, and compares the ratio of the target element to the standardized element with the ratio of two elements’ baseline value in the reference area to produce the enrichment factor that can be compared among different elements. There is no corresponding standard for the determination and selection of the baseline value in this method, which results in different outcomes in practical application.

The risk assessment code method (RAC) is based on the different binding forces of heavy metals and metalloids in soil, which provides a new way for developing ecological risk assessment. This method considers the exchangeable and carbonate-bound states, that is, so-called acid extractable state, as the bioavailable part of heavy metals and metalloids, and evaluates the bioavailability of heavy metals and metalloids in soil by calculating the percentage of the acid extractable fraction of the total amount. The higher the bioavailability, the greater the risk to the environment.

All the above four methods can only evaluate the pollution degree or ecological risk of a single element, and cannot be applied to evaluate the cumulative pollution from various elements. The Nemerow index, Pollution Load Index (PLI), and the potential ecological risk index were aimed at solving this problem.

The Nemerow index covers all single pollution indices of elements involved in evaluation, and highlights the weights of highest concentrations of single pollution in the assessment results, then it can avoid the average effect to weaken their weights, but at the same time may exaggerate the impacts of maximum values or some outlier values, thereby reducing the sensitivity of this method. Moreover, the application of the maximum values of single pollution indices does not have the basis of ecotoxicology. Another drawback of the Nemerow index is that it cannot eliminate the regional differences in the background values, which may cause inconvenience for interregional comparison.

PLI can directly reflect the contribution degree of each element to comprehensive pollution, but it cannot reflect its chemical activity and bioavailability, and the background differences caused by different pollutant sources are not under consideration.

The potential ecological risk index method can link the ecological effects, environmental effects and toxicology of heavy metals and metalloids, and also can make the risk level of different elements be reflected in the evaluation, but the approach concerned only limnic systems when Hakanson created it. When applied to soil, there is no characteristic index to reflect the toxic effects of soil’s physical and chemical properties on heavy metals and metalloids if the model is not corrected, which could produce insignificant and even unreasonable results.

With respect to the disadvantage of the Nemerow index, PLI and the potential ecological risk index, Chen et al. proposed the comprehensive pollution index (CPI), which takes into consideration the valence state effect of elements, environmental quality standard, element background value and specific soil load capacity [23]. CPI has been recommended in Technical Specification for Soil Environmental Monitoring, which is one of the occupation standards of environmental protection in People’s Republic of China [24].

After analyses and comparisons, for discrete samples, we selected three methods to assess heavy metal and metalloid pollution: (1) Contamination factor (CF), to assess whether a single element exceeds the background value and environmental quality standards. (2) Risk assessment code (RAC), to evaluate the ecological risk of the key pollutant. (3) Comprehensive pollution index (CPI), to evaluate the compound pollution degree of five target elements.

#### 2.2.2. Comprehensive Pollution Index (CPI)

Early studies showed that ionic impulsion can be used as a comprehensive indicator of heavy metal and metalloid pollution [25], which is a parameter related to the concentration of plant elements, being expressed as:

(1) $$I=\sum C_{i}{}^{\frac{1}{n_{i}}}$$

where *C_i_* is the concentration of element *i* in the plants (dry weight, mmol g^−1^), *n_i_* is the oxidation number of element *i*.

Under normal conditions, ionic impulsion is an approximate constant. However, it increases with the increase in poisonous element concentration in soil, which makes it is possible to use this parameter to evaluate the degree of pollution. On the basis of plant ionic impulsion, Chen and Zheng expanded the relationship between plant ion impulse and heavy metal and metalloid ion impulse and proposed the relative pollution equivalent (RPE) [26], the oxidation number of elements and their corresponding toxicity being considered, to reflect the relative influence of different elements in soil. In addition, another two parameters were introduced—deviation degree of measured concentration from the background value (DDMB), and deviation degree of soil standard from the background value (DDSB) [23]. DDMB can quantize how much the target heavy metals and metalloids exceed local background values but still less than the standard values of the environmental quality or the starting value of the pollution. DDSB is a measure of the load capacity of the local soil environment, showing its buffer capacity for heavy metals and metalloids and other pollutants.

The three parameters are calculated through Formulae (2)–(4):

(2) RPE=[∑i=1N(Ci/CSi)1ni]/N

(3) DDMB=[∑i=1N(Ci/CBi)1ni]/N

(4) DDSB=[∑i=1N(CSi/CBi)1ni]

where *C_i_*, *C_iS_*, and *C_iB_* are the element i’s concentration measurement, standard and background value, respectively; *n_i_* is the oxidation number of element *i*; and N is the number of assessed elements. As the relationship between oxidation number and its toxicity has been taken into account when Environmental Quality Standard for Soils are set, the stable state of the elements in the soil is generally adopted in the actual assessment. For example, the oxidation number of arsenic is 5, and chromium is 3 [27].

The CPI is determined by Formula (5):

(5) CPI=X·(1+RPE)+Y·DDMB/DDSB

where X, and Y stand for the numbers which the measured heavy metal and metalloid concentrations beyond the limits of standards and background values, respectively.

## 3. Contamination Assessments Based on Discrete Sampling Points

### 3.1. General Characteristics of Heavy Metal and Metalloid Contaminations in the Soil

The descriptive statistical summary of five target elements’ concentrations in soil samples is shown in Table 1.

### 3.2. Soil Contamination Assessments based on the Contamination Factor (CF) Method

In this study, we calculated two kinds of contamination factors (*CF*), *CF*_1_ was for assessing the accumulation effect of single target element, and *CF*_2_ was for showing whether the target element exceeded the standard. *CF* values were determined by (6) and (7):

(6) CF1=Ctarget element CB

(7) CF2=Ctarget element CS

where *C_target_*_*element*_, *C_B_* and *C_S_* are measured target element concentrations of samples, the background value of the study area and environmental quality standard for soil, respectively. The results of target element contamination by means of contamination factors are illustrated in Table 2. From Table 2, it may be interpreted that most U, As, Pb and Cr in soil were mainly extraneous, since the percentages of *CF*_1_ > 1 reached, respectively, 98.54%, 72.19%, 60.49% and 67.32%.

The contamination levels may be classified based on *CF*_2_s’ quantities, *CF*_2_ ≤ 1: unpolluted; 1 < *CF*_2_ ≤ 2: slightly polluted; 2 < *CF*_2_ ≤ 3: moderately polluted; and *CF*_2_ > 3: strongly polluted. Fortunately, benefiting from the load capacity of the local soil environment, the percentages of polluted samples were relatively low, which were 13.18%, 1.47%, 1.95%, 1.95% and even 0% for U, Mn, As, Pb and Cr, respectively. We deduced that the abandoned uranium tailings pond did contaminate the surrounding soil and especially, we should pay attention to the contamination of the U element. In fact, it was possible for the contamination of U to be subordinate in study analogies. For instance, in the vicinity of a cement plant and a former open-cast uranium mine in Central Argentina, the researchers found that the mean total Ba concentration exceeded soil quality guidelines for residential areas, with the maximum total As and Co concentrations surpassing the agricultural and residential limits stated in national and international legislations [5]; the assessment results by RI showed that the ecological risk of heavy metals in the farmland soil surrounding a uranium tailings pond was high, but the main factor that caused the ecological hazard was cadmium, followed by Hg and As [12].

**Figure 2:**
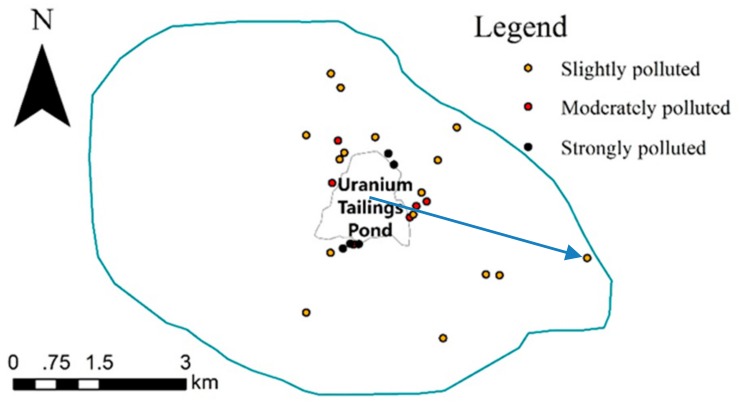
The distribution of the 27 samples whose U’s *CF*_2_ > 1.

Figure 2 illustrates the distribution of the 27 samples whose U’s *CF*_2_ > 1, from which it can be seen that the 6 moderately polluted and 5 strongly polluted samples were immediately near the uranium tailings pond and the 16 slightly polluted samples were scattered around the pond, with the farthest distance away from the center (which the arrow indicated) being 4088 m.

### 3.3. Risk Assessment Codes (RAC) of Uranium Element

**Figure 3:**
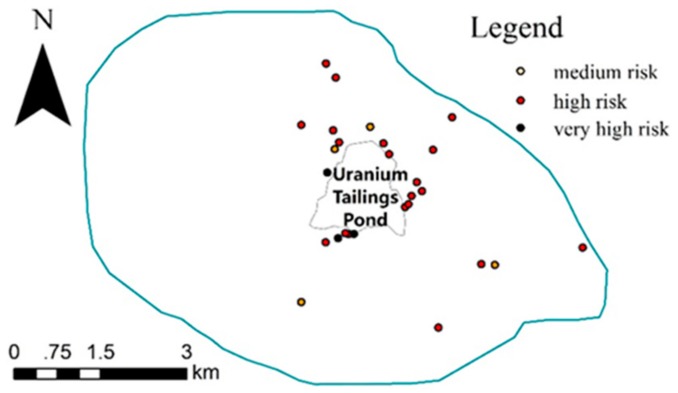
The distribution of the 27 samples’ RAC.

To evaluate their environmental risks, it was necessary to calculate and analyze risk assessment codes (RAC) of uranium element for the 27 samples whose total concentrations of U’s various chemical form reached the polluted level. We extracted the acid-soluble fractions (F1) of the 27 samples with the BCR sequential extraction method (BCR-SEP) and tested the uranium contents. Uranium’s RAC of the 27 samples, based on F1 percentage: % F1 < 1, no risk; % F1 = 1–10, low risk; % F1 = 11–30, medium risk; % F1 = 31–50, high risk; % F1 > 50, very high risk [33] are listed in Table 3 and the spatial distribution is shown in Figure 3.

From Table 3 and Figure 3, we understood that 4 samples were medium risk, 20 samples were high risk and 3 were very high risk, among which the 3 very high-risk samples were just near the borderline of the tailings pond. The uranium was relatively more easily bioavailable in this study when compared with similar ones, for example, the acid-soluble fraction of U in the soil samples from a uranium mill tailing pond in northwest China was only 1.6% [8].

It was reported that lettuce bioconcentration is more related to available uranium species in water than to its uranium concentration [3]. In an earlier study, we found that wild ramie in this tailings pond had strong uranium bioconcentration and transfer capacities, but when we carried out a pot experiment, we did not obtain satisfactory results [34]. We analyzed the possible reasons in that paper, but we did not realize the difference in bioavailabilty of U was one of factors which affected ramie’s bioconcentration and transfer capacities. Undoubtedly, easier bioavailabilty means higher health risk to the living creatures inhabiting the area; on the other hand, we speculated the likelihood of bioremediation of this kind of contaminated soil.

### 3.4. Soil Pollution Assessments Based on the Comprehensive Pollution Index (CPI) Method

We calculated the CPI of all 205 soil samples and listed these in Table 4, and found 174 samples were unpolluted but invasive and accumulated, 1 slightly polluted, 24 moderately polluted and 6 strongly polluted according to the classification standards in Table 5.

**Figure 4:**
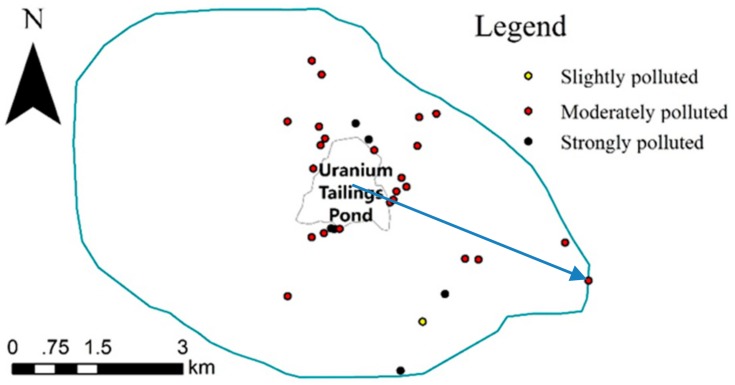
The distribution of the 31 samples whose CPI ≥ 1.

As for the spatial distribution of the 31 polluted samples shown in Figure 4, the 31 samples were in the east part of the study area, being similar to the distribution of 27 uranium polluted samples, since uranium was the highest pollution element. By contrast, both the number of samples and distribution scope were greater. The farthest distance away from the center reached 4948 m (which the arrow indicated), and the spatial correlation to the tailings pond was weaker, when the 5 target elements’ comprehensive pollution was compared with single uranium pollution.

## 4. Pollution Spatial Continuous Distribution Mapping and Variation Characteristics

In this section, we only took into account *CF*_2_ of U and CPI, since the single pollution effects of Mn, Pb, As, and Cr can be neglected according to Table 2.

### 4.1. Spatial Interpolation and Mapping of Pollution Continuous Distribution

Spatial interpolation, which includes deterministic methods and geostatistics, can transform the measured data of discrete sampling points into continuous data surfaces. The deterministic interpolation methods, such as inverse distance weighted interpolation (IDW), trend surface method, and spline function method, are based on the similarity between sampling points or the smoothness of the entire surface to create the fitting surface. By using the spatial structure of original data and semivariogram, geostatistics conducts agonic estimation of the regionalized variables of the sampling area [36,37]. Geostatistics, being proven to be one of the most effective methods to analyze the spatial distribution characteristics and variation law of soil [38,39,40,41,42], can shift the assessments of individual sampling points to the study of variation pattern and spatial distribution of the whole sampling area. Kriging interpolation, which is based on geostatistics, not only considers the distance between the sampled points and the un-sampled points, but also takes into account the spatial distribution of the sampled points and the spatial azimuth relation of the un-sampled through the variational function and structural analysis [43]. Goovaerts (1992) [44] applied Kriging analysis for the first time to the study of soil, and achieved satisfactory research results. Even across the whole United States, geostatistics can show the soil’s variation of spatial variability and properties very well [45].

**Figure 5:**
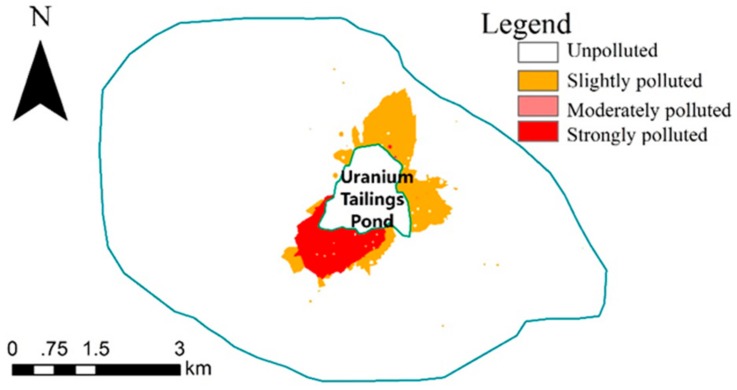
The continuous distribution of U’s *CF*_2._

**Figure 6:**
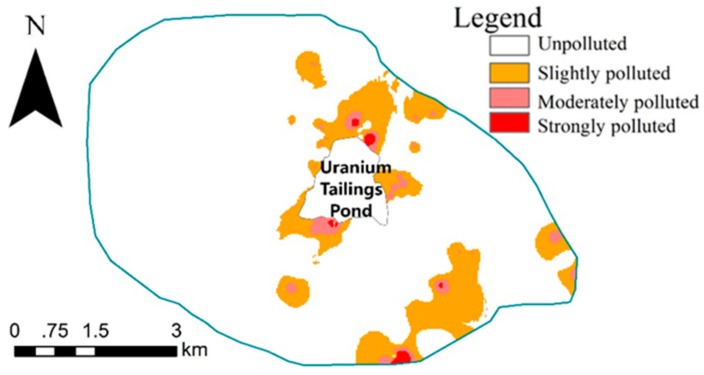
The continuous distribution of CPI.

However, ordinary Kriging requires that data fit a normal distribution and regionalized variables meet the second-order stationary hypothesis. In practice, the hypothesis is often not supported, that is, there is a drift phenomenon, when data need to be processed to follow a normal distribution to meet the theoretical requirements of geostatistics. At present, there are three kinds of data transformation methods: logarithmic transformation, Box-Cox transformation and Johnson transformation. Among the three methods, the Johnson transformation [46] includes 3 complex transformation curves and has more powerful transformation capability. It has been widely used in recent years [47,48], and its transformation success rate is greater than that of logarithmic transformation and Box-Cox transformation [49,50,51]. In this paper, we found that both the contamination factor of U and CPI did not conform to a normal distribution. The Johnson transformation did help to realize the normalization of *CF*_2_ of U, but not CPI. Therefore, we obtained the continuous distribution maps, as shown in Figure 5 and Figure 6, spatially interpolated by ordinary Kriging and Inverse Distance Weighted (IDW), respectively. Table 6 indicates the statistical areas and percent of uranium pollution degree and comprehensive pollution degree based on Figure 5 and Figure 6.

According to Figure 5 and Figure 6 and Table 6, of the 41.25 km^2^ study area, 3.14 km^2^ was polluted by uranium and 5.63 km^2^ was polluted comprehensively by 5 target elements. Moderately polluted zones were relatively small. Strong U pollution was mainly situated on the southwest of the tailings pond, and a slightly U-polluted zone occurred mainly to the north and east. Strongly and slightly comprehensively polluted zones spread diffusively in the east of the study area.

### 4.2. Pollution Spatial Variation Characteristics

Geostatistics introduces a powerful tool, the variogram, which can reflect the spatial variation characteristics and structure of regionalized variables.

**Figure 7:**
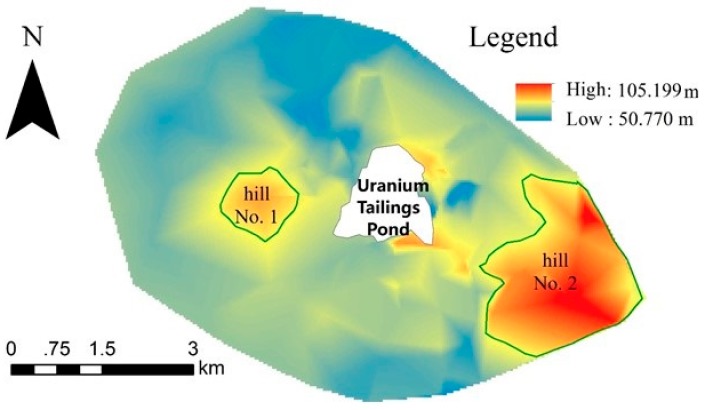
Digital Elevation Model of the study area.

GS + 9 geostatistics software was used to calculate the best variogram model in the principle of determination coefficients (r^2^) being maximum and residuals sum of squares (RSS) being minimum. Table 7 lists the optimal variogram theoretical models and the related parameters of U’s *CF*_2_ and CPI. The nugget to sill ratio of U’s *CF*_2_ was 8.08%, showing that there was high spatial dependence. Strongly spatially dependent properties may be controlled by intrinsic variations in soil characteristics, such as climate, topography and soil types, etc. [52]. We found that the U-polluted zones coincided basically with the basins which were to the southwest, north and east of the tailings pond, while further than hill No. 1 from the tailings pond there was no uranium polluted at all, and near hill No. 2 there were only very small uranium polluted spots, referring to the Digital Elevation Model of the study area which was mapped in Figure 7. Therefore, it was deduced that the spatial variation of uranium was in close relationship with local topography, and probably precipitation was the driving force of uranium pollution diffusion. On the other hand, the nugget to sill ratio of CPI was as high as 84.4%, which meant that CPI had weak spatial dependence with random characteristic. It is necessary to perform denser sampling in the study area to reflect the spatial variation structure of CPI.

## 5. Conclusions

In view of the characteristics of pollutants inside the uranium tailings pond and by reference to the Environmental Quality Standard for Edible Agricultural Products of China (HJ/T 332-2006), 4 heavy metals and 1 metalloid (U, Mn, As, Pb, Cr) were targeted in this study. The descriptive statistics, contamination factor analyses of 5 target elements concentrations according to the soil samples indicated that U pollution near the tailings pond was fairly serious, even after it was decommissioned for 20 years, and being the key pollutant, U’s ecological risk was relatively high, inferring from RAC analysis.

The spatial distribution mapping and geostatistical variograms illustrated that uranium accumulated near the tailings pond, especially to its southwest, east and north, and its distribution was closely related to the local topography. We believe that precipitation was the driving force of uranium pollution diffusion. Geostatistical and topographic analyses, whose results coincided with U’s relatively high acid extractable state extract proportions, can be an effective way to research the mobility of contaminants.

The other 4 elements’ single pollutions were less serious than that of U, but the area of 5 target elements’ comprehensive pollution zone was larger than that of U pollution and cannot be ignored according to the Comprehensive Pollution Index (CPI) assessment results. It was deduced that potentially, there were other pollution sources besides the uranium tailings pond, since CPI exhibited weak spatial dependence with random characteristics.

Obviously, the study area is not fit for cultivation. It is suggested that the pond managers and policy makers enhance monitoring and take some emergency measures to fight against U-pollution. We recommend bioremediation as one of choices based on U’s bioavailability. Moreover, special alerts should continue to search for other potential pollution sources.

## Figures and Tables

**Table 1:** Descriptive statistical summary of five target element concentrations in soil samples.

Statistical Quantity	U	Mn	As	Pb	Cr
Mean (mg/Kg)	13.69	398.27	17.56	31.09	71.90
Standard deviation(mg/Kg)	14.48	270.58	7.25	9.44	8.94
Maximum (mg/Kg)	120.52	1603.28	66.68	60.38	96.43
Minimum (mg/Kg)	3.21	104.01	6.17	7.16	48.39
Skewness	13.16	8.66	12.64	2.84	0.11
Kurtosis	181.68	98.03	172.14	12.91	12.45
Local background value *	4.2	441	14	27	68
Environmental quality standard for soils **	15.5	1500	40	80	150

Notes: * Local background values were from Soil Background Values and Its Research Methods in Hunan Province, China. Beijing, China Environmental Science Press [28]. ** There are three origins of environmental quality standards: (1) The Grade Ⅱ standard in Environmental quality standard for soils (GB 15618–1995) [29], which specifies the restriction values to protect agricultural production and maintain human health for eight kinds of heavy metal and metalloids including arsenic, lead and chromium; (2) Technical regulations on the assessment of soil pollution status in China [30], where we found the restriction value for manganese. (These first two kinds of standard values are equivalent to preliminary remediation goal in America or soil guideline values in British [31].) (3) The paper Contribution for the Derivation of a Soil Screening Value (SSV) for Uranium, Using a Natural Reference Soil [32], which offered a reference restriction value for uranium. We could not find any intervention value of U, so we had to adopt this soil screening value.

**Table 2:** The results of target element contaminations by means of contamination factors.

Target Element	*CF*_1_ ≤ 1(%)	*CF*_1_ > 1(%) (Exogenous Invasion)		Contamination Percentage by Means of Contamination Factor (%)		
		*C_target_*_*element*_ ≤ *C_S_*	*C_target_*_*element*_ > *C_S_*	Slightly Polluted 1 < *CF*_2_ ≤ 2	Moderately Polluted 2 < *CF*_2_ ≤ 3	Strongly Polluted *CF*_2_ > 3
U	1.46	85.36	13.18	7.80 *	2.93 *	2.44 *
Mn	70.24	28.29	1.47	0.98	0	0.49
As	27.81	70.24	1.95	1.46	0	0.49
Pb	39.51	58.54	1.95	1.95	0	0
Cr	32.68	67.32	0	0	0	0

Note: Strictly, these values with * could not be named as polluted percentages before environmental risk evaluation, since we adopted the soil screening value as the baseline value to calculate U’s *CF*_2_.

**Table 3:** Acid-soluble fraction (F1), %F1 and uranium’s RAC of the 27 samples.

Soil Sample	Acid-Soluble Fraction (F1) (mg/Kg)	Total U (mg/Kg)	Percentage of the total U (% F1)	Risk Assessment Code (RAC)
No.001	13.30	33.15	40.13	high risk
No.002	9.35	30.69	30.47	high risk
No.003	13.71	40.81	33.59	high risk
No.004	8.33	17.72	46.99	high risk
No.011	14.42	34.24	42.11	high risk
No.021	4.78	19.25	24.85	medium risk
No.023	6.03	17.20	35.04	high risk
No.033	15.46	41.14	37.59	high risk
No.035	55.29	102.68	53.85	very high risk
No.050	7.73	20.03	38.61	high risk
No.051	33.51	62.37	53.72	very high risk
No.052	235.39	726.96	32.38	high risk
No.060	5.39	15.87	33.95	high risk
No.068	13.17	34.62	38.04	high risk
No.070	5.41	15.94	33.94	high risk
No.071	6.38	24.49	26.05	medium risk
No.074	17.65	33.92	52.03	very high risk
No.083	2.41	20.45	11.77	medium risk
No.086	40.00	120.52	33.19	high risk
No.087	19.17	60.21	31.85	high risk
No.098	6.49	16.37	39.62	high risk
No.106	7.20	15.77	45.66	high risk
No.115	6.93	18.37	37.73	high risk
No.131	9.49	19.21	49.39	high risk
No.141	7.28	15.68	46.44	high risk
No.168	8.43	17.12	49.22	high risk
No.201	4.19	22.12	18.96	medium risk

**Table 4:** Descriptive statistical summary of soil pollution comprehensive assessments.

Statistical Quantity	X	Y	RPE	DDMB	CPI	CPI > 1
Mean	0.19	3.28	0.74	1.04	0.82	2.93
Standard deviation	0.49	1.10	0.07	0.12	1.05	1.37
Maximum	3	5	1.10	1.68	7.38	7.38
Minimum	0	1	0.43	0.64	0.09	1.86
Skewness	3.18	−0.31	1.47	1.90	3.50	2.53
Kurtosis	11.83	−0.50	10.12	10.20	15.66	5.98

**Table 5:** Classification standards of the Comprehensive Pollution Index (CPI).

X	Y	CPI	Comprehensive Pollution Assessments	Number of Sampling Points and the Corresponding Percent	
0	0	0	Background state	0	0
0	≥1	0 < CPI < 1	Unpolluted but invaded and accumulated	174	84.9%
≥1	≥1	1 ≤ CPI < 2	Slightly polluted	1	0.5%
		2 ≤ CPI < 3	Moderately polluted	24	11.7%
		CPI ≥ 3	Strongly polluted	6	2.9%

Note: The comprehensive pollution assessment standard was mainly from these two references [24,35], and refined by the pollution degree by the authors, which had been classified as polluted generally when CPI ≥ 1.

**Table 6:** The areas and percent of uranium pollution degree and comprehensive pollution degree.

Uranium Pollution Degree	Area (km^2^)	Percent (%)	Comprehensive Pollution Degree	Area (km^2^)	Percent (%)
Unpolluted	38.11	92.38	Unpolluted	35.62	86.34
Slightly polluted	2.02	4.91	Slightly polluted	4.90	11.87
Moderately polluted	0.01	0.04	Moderately polluted	0.60	1.46
Strongly polluted	1.11	2.67	Strongly polluted	0.13	0.33
∑	41.25	100	∑	41.25	100

**Table 7:** Theoretical models and parameters of the variogram of *CF*_2_ of U and CPI.

Pollution Index	Theoretical Model	Nugget (C_0_)	Sill (C_0_ + C)	Nugget to Sill Ratio [C_0_/(C_0_ + C)]	Range (m)	RSS	r^2^
*CF*_2_ (U)	Gaussian	0.082	1.015	8.08%	328	0.019	0.306
CPI	Exponential	0.178	1.138	84.4%	132	0.377	0.224
